# Sedation and General Anesthesia in Non-Cooperative Dental Patients: An Italian Clinical Experience

**DOI:** 10.3390/jcm15072532

**Published:** 2026-03-26

**Authors:** Giulio Cirignaco, Giorgio Lo Giudice, Angela Rosa Caso, Marco Gasperoni, Simone Clementi, Luca Gentili, Marco Messi, Stefania Troise, Luigi Angelo Vaira, Roberto Lo Giudice, Giuseppe Consorti

**Affiliations:** 1 Department of Medicine, Section of Maxillo-Facial Surgery, University of Siena, Viale Bracci, 53100 Siena, Italy; g.cirignaco@student.unisi.it (G.C.); angelarosacaso@gmail.com (A.R.C.); m.gasperoni@student.unisi.it (M.G.); 2 Division of Maxillofacial Surgery, Department of Neurological Sciences, Marche University Hospitals-Umberto I, 60126 Ancona, Italy; 3 Department of Medicine and Surgery, University of Enna “Kore”, Piazza dell’Università, 94100 Enna, Italy; giorgio.logiudice@unikore.it; 4 Private Practice, 63100 Ascoli, Italy; simoneclementi250798@gmail.com; 5 Department of Dentistry, Engles Profili Hospital of Fabriano, 60044 Fabriano, Italy; luca.gentili@sanita.marche.it (L.G.); marco.messi@sanita.marche.it (M.M.); 6 Maxillofacial Surgery Unit, Department of Neurosciences, Reproductive and Odontostomatological Sciences, University of Naples Federico II, 80138 Naples, Italy; stefy.troise@gmail.com; 7 Maxillofacial Surgery Operative Unit, Department of Medicine, Surgery and Pharmacy, University of Sassari, Viale San Pietro 43/B, 07100 Sassari, Italy; lavaira@uniss.it; 8 Department of Biomedical and Dental Sciences and Morphological and Functional Imaging, University of Messina, 98125 Messina, Italy; roberto.logiudice@unime.it; 9 Department of Clinical Specialistic and Dental Sciences, Marche Polytechnic University, 60126 Ancona, Italy

**Keywords:** non-cooperative patients, dental sedation, conscious sedation, deep sedation, general anesthesia, special care dentistry, access to dental care

## Abstract

**Background**: Dental care for non-cooperative patients is a major clinical and organizational challenge, particularly in individuals with intellectual or neurodevelopmental disabilities and in patients with severe dental anxiety or phobia. When behavioral techniques are insufficient, conscious or deep sedation or general anesthesia may be required, but practical guidance on selection and care pathways remains fragmented. **Methods**: We combined a retrospective observational analysis from a single Italian academic center with a narrative review of the international literature. Forty-one sedation-assisted dental sessions were included. Demographics, indication for non-cooperation, sedation regimens, procedures, completion rates, and adverse events were descriptively analyzed. **Results**: The cohort included pediatric and adult patients; non-cooperation was mainly related to disability/neurodevelopmental conditions or severe dental phobia. Benzodiazepine-based oral or intravenous sedation, sometimes combined with low-dose propofol, enabled completion of all planned procedures without major adverse events or conversion to general anesthesia. The literature supports general anesthesia for profound non-cooperation or extensive treatment needs, but availability and waiting lists limit access; sedation is effective for selected cases with appropriate organizational support. **Conclusions**: An individualized stepped-care model integrating behavioral management, sedation, general anesthesia, and structured preventive recall may optimize access and outcomes within the Italian context and strengthen long-term post-treatment attendance.

## 1. Introduction

Dental treatment in non-collaborative patients remains a major challenge in daily clinical practice and represents a relevant public health issue. A lack of cooperation may be related to severe dental anxiety or phobia, intellectual disability, neurodevelopmental disorders such as autism spectrum disorder, psychiatric conditions, or previous traumatic dental experiences, often preventing the completion of dental procedures under local anesthesia alone [1,2,3,4,5].

Therefore, these patients frequently experience delayed access to care, incomplete treatment, and progressive deterioration of oral health, with significant implications for quality of life and long-term outcomes [6].

First-line management of anxiety and mild behavioral problems relies on non-pharmacological techniques such as Tell–Show–Do, positive reinforcement, visual supports, and environmental adaptations (e.g., reduced sensory stimuli, quiet rooms). These strategies can improve cooperation in some patients, particularly children with milder conditions. However, for many individuals with profound behavioral dysregulation or complex disabilities, behavioral techniques alone are insufficient. In such cases, dentists must consider pharmacological approaches ranging from minimal and moderate sedation to deep sedation and general anesthesia (GA) [7,8].

In this context, pharmacological behavior management plays a crucial role in enabling dental care. Conscious and deep sedation techniques have been increasingly adopted to facilitate treatment in non-collaborative patients by reducing anxiety, improving compliance, and allowing the execution of procedures while maintaining spontaneous ventilation and protective reflexes [9,10]. Compared with general anesthesia, sedation may offer advantages in terms of reduced invasiveness, faster recovery, and lower organizational burden when appropriately indicated and performed by trained teams [11].

Sedation and GA markedly expand what can be achieved in non-cooperative patients—enabling full examinations, radiographs, restorative dentistry, and oral surgery—but they require specific expertise, monitoring, and infrastructure, and they increase costs and resource use. Access to these modalities is uneven. In Italy, specialized units capable of providing dental care under GA or deep sedation are concentrated in a limited number of centers, and waiting lists are often long. Many patients receive care only when pain or infection becomes intolerable, at which point comprehensive rehabilitation under GA is frequently the only realistic option [12,13]. Patients with neurodevelopmental disorders and intellectual disabilities represent a particularly vulnerable subgroup. Previous studies have shown that these patients often require advanced behavior management strategies to access dental care, with sedation frequently considered a valuable alternative to general anesthesia for selected procedures [14,15].

However, the effectiveness of sedation in this population is highly variable, and unsuccessful sedation attempts have been reported, highlighting the importance of careful patient selection, individualized protocols, and realistic expectations regarding treatment feasibility [16]. Severe dental anxiety and phobia also constitute a major indication for sedation-assisted dental care in both pediatric and adult populations. Dental anxiety has been consistently associated with avoidance behaviors, irregular dental attendance, and increased treatment needs, further complicating clinical management [17,18]. In such cases, sedation has been reported to improve treatment acceptance and completion, although evidence supporting specific pharmacological regimens remains limited and largely based on observational studies [19].

Despite the widespread clinical use of sedation in dentistry, the available scientific evidence remains heterogeneous. Most published studies consist of retrospective case series, service evaluations, or narrative reviews, with substantial variability in patient characteristics, sedation protocols, outcome measures, and reporting standards [20,21,22,23,24,25,26].

Consequently, there is a lack of robust comparative data to guide clinicians in selecting optimal sedation strategies or defining standardized care pathways for non-collaborative patients.

Within this framework, real-world clinical studies remain essential to describe current practice, explore feasibility and short-term safety, and identify gaps in the existing literature. Therefore, the aim of the present study was to describe the clinical characteristics, sedation protocols, and short-term outcomes of dental treatments performed under conscious or deep sedation in non-collaborative patients, and to contextualize these findings within the current scientific literature on sedation-assisted dental care.

## 2. Materials and Methods

This retrospective observational study was conducted at a single academic center and involved the review of consecutive dental treatment sessions performed under conscious or deep sedation in non-collaborative patients.

Patients were identified through clinical and administrative records over the study period. Inclusion criteria were age ≥ 6 years and inability to undergo dental treatment under local anesthesia alone due to behavioral, cognitive, or psychological factors. Exclusion criteria included incomplete documentation of sedation protocols or procedures planned under general anesthesia. Two treatment sessions initially intended for sedation required alternative management strategies, including intramuscular sedation or conversion to general anesthesia, and were excluded from the primary descriptive analysis but reported separately for completeness. Studies focusing solely on paediatric routine care without special needs, opinion pieces, and case reports were excluded, and the literature search was censored at 1 September 2025.

In addition to descriptive reporting, exploratory comparisons were performed between sessions conducted for disability/neurological impairment and those conducted for severe dental phobia.

Patients categorized in the ‘severe dental phobia’ group were referred to our hospital with a diagnostic assessment performed outside our institution, typically within public outpatient services (local health services, ASL) and/or by community specialists in clinical psychology and/or psychiatry. Referral documentation and standardized clinical forms accompanying the patient explicitly reported severe dental anxiety/phobia and indicated pharmacologic management (sedation and/or general anesthesia) due to inability to undergo dental treatment under local anesthesia. For this retrospective analysis, the definition of severe dental phobia was based on the presence of this specialist referral documentation in the medical record.

Sedation was defined according to internationally accepted clinical criteria as a drug-induced depression of consciousness during which patients retained spontaneous ventilation and protective reflexes and remained responsive to verbal commands or light tactile stimulation [9,21]. Conscious and deep sedation techniques were selected on the basis of patient characteristics, behavioral profile, and procedural complexity, in line with published dental sedation frameworks [23].

Pharmacological agents commonly employed included oral and intravenous benzodiazepines, alone or in combination with short-acting agents. Midazolam was the primary sedative used due to its favorable pharmacokinetic profile and widespread acceptance in dental sedation practice [22]. In selected cases, low-dose propofol or opioid adjuncts were administered to achieve adequate sedation depth while maintaining patient safety, as described in the dental and anesthesiology literature [20,27,28].

Pre-sedation assessment included medical and behavioral history, identification of potential contraindications, and baseline vital signs. Information regarding ASA physical status and detailed medical comorbidities was inconsistently documented in routine clinical records and therefore was not included in the quantitative analysis. Intraoperative monitoring followed established clinical guidelines and included continuous pulse oximetry, heart rate, blood pressure, and respiratory rate, with supplemental oxygen administered as clinically indicated. Reversal agents, including flumazenil, were readily available and administered when required.

Sedation depth was titrated to achieve patient cooperation while preserving safety margins, consistent with definitions and recommendations in dental sedation literature. Procedural documentation included sedative agents and dosages, level of sedation achieved, completion of planned dental treatment, and occurrence of any adverse events or sedation-related interventions. Patients were monitored in a dedicated recovery area until they met discharge criteria, including stable vital signs and return to baseline cognitive function. During the preparation of this manuscript/study, the author(s) used ChatGPT-5 (OpenAI, San Francisco, CA, USA) for the purposes of proofreading and language refinement of this manuscript. The authors have reviewed and edited the output and take full responsibility for the content of this publication.

### Statistical Analysis

The sedation-assisted dental treatment session was considered the unit of analysis, including repeated sessions in the same patient when applicable. Continuous variables are reported as mean ± standard deviation (SD) and range, and categorical variables as counts and percentages. For key proportions (e.g., treatment completion and zero-event safety outcomes), exact 95% confidence intervals (Clopper–Pearson) were calculated to reflect estimate precision in a small sample.

For inferential analyses, two-sided tests were applied with a significance threshold set at α = 0.05. Mean age was compared between sessions performed for disability/neurological impairment and those performed for severe dental phobia using Welch’s *t*-test (allowing unequal variances). Differences in the distribution of procedure categories between groups were assessed using Pearson’s chi-square test; when clinically relevant, 2 × 2 contrasts were explored using Fisher’s exact test and expressed as relative risks (RR) with 95% confidence intervals. Effect sizes were reported as Hedges’ g for continuous comparisons and Cramer’s V for contingency tables.

## 3. Results

A total of 43 sedation-assisted dental treatment sessions were identified during the study period. Of these, 41 sessions met the inclusion criteria and were included in the primary descriptive analysis, while two sessions initially planned under sedation required alternative management strategies (intramuscular sedation or general anesthesia) and were excluded from the main analysis.

In addition to descriptive reporting, exploratory subgroup comparisons were performed between sessions conducted for disability/neurological impairment and those conducted for severe dental phobia. The final sample consisted of 41 treatment sessions performed in non-collaborative patients. Twenty sessions (48.8%) involved patients with neurodevelopmental or intellectual disabilities, including autism spectrum disorder, Down syndrome, and cognitive impairment, while 21 sessions (51.2%) were performed in patients with extreme dental anxiety or severe non-cooperation (Table 1).

The overall mean age of the study population was 32.0 ± 14.0 years (range, 6–75). Patients in the disability/neurological impairment group were older on average than those treated for severe dental phobia (35.4 ± 12.8 vs 28.6 ± 14.2 years), corresponding to a mean difference of 6.8 years (95% CI −1.7 to 15.3; Welch’s *p* = 0.115) and a moderate standardized effect size (Hedges’ g ≈ 0.49).

### 3.1. Sedation Modalities and Protocols

Sedation was delivered using different routes of administration depending on patient characteristics and procedural requirements (Table 2).

The route of administration was documented in 39 of the 41 included sessions (95.1%). Among sessions with documented route, oral sedation alone was used in 12/39 (30.8%), intravenous sedation alone in 18/39 (46.2%), and a combined oral–intravenous approach in 9/39 (23.1%).

In selected complex cases, IV midazolam was combined with low-dose propofol to achieve a deeper “twilight” state while maintaining spontaneous respiration. Inhalation sedation with nitrous oxide was not used in this series, either because patients’ level of non-cooperation made mask placement impractical or because equipment was not available in the participating centers.

Adjuvants: Flumazenil was administered in specific instances to accelerate recovery and facilitate same-day discharge.

### 3.2. Dental Procedures Performed

A wide range of dental procedures was successfully completed under sedation (Table 3).

Preventive procedures, including professional oral hygiene and scaling, accounted for 19 sessions (46.3%), with a higher prevalence in patients with disabilities (14 sessions) compared with those with dental phobia (5 sessions).

The sedative regimens and adjunctive agents used across the analyzed sessions are summarized in Table 4.

Restorative procedures, such as fillings and endodontic treatments, were performed in 12 sessions (29.3%), predominantly in patients with dental phobia (9 sessions). Surgical procedures, including simple and surgical extractions and germectomies, were carried out in 10 sessions (24.4%), with a greater proportion observed in the phobia group (7 sessions) compared with the disability group (3 sessions). No sessions were limited to diagnostic procedures alone.

In exploratory analyses, the distribution of procedure categories differed significantly between indication groups (Pearson χ^2^(2) = 8.84, *p* = 0.012; Cramer’s V = 0.46), driven mainly by a higher proportion of preventive procedures in disability-related sessions (14/20) compared with phobia-related sessions (5/21; RR = 2.94, 95% CI 1.30–6.66; Fisher’s *p* = 0.0048). Detailed subgroup comparisons, including effect sizes and relative risks, are reported in Table 5.

All dental procedures included in the primary analysis were completed as planned under sedation, without the need for treatment interruption or escalation of care.

### 3.3. Clinical Outcomes and Completion Rates

All 41 sessions (100%) resulted in the completion of the planned procedures without the need for conversion to general anesthesia (GA). No major adverse events were recorded. Specifically, there were no episodes of respiratory depression, cardiovascular instability, emergency airway management, or treatment interruption. Minor events were limited to transient agitation or delayed recovery, which were managed intraoperatively without clinical consequences. Flumazenil was available and administered when clinically indicated to facilitate recovery. All patients were monitored in a dedicated recovery area and discharged on the same day after meeting standard discharge criteria, including stable vital signs and return to baseline cognitive status.

Scope of Care: Treatments were largely focused on professional hygiene (scaling, plaque and calculus removal); Restorative treatments (fillings for dental caries); Selected surgical procedures (extractions of non-restorable teeth or residual roots, occasional removal of impacted teeth).

However, the scope of each session was often limited to specific quadrants due to the practical time constraints of ambulatory sedation. In rare cases, diagnostic imaging (e.g., intraoral radiographs) could not be obtained in awake patients.

All procedures planned for each individual session were completed in all 41 sessions. Importantly, in non-cooperative patients, the overall treatment plan was intentionally delivered in a staged, stepwise manner: most sessions were designed with a limited scope (e.g., a single quadrant or a restricted number of teeth) to maintain safety, tolerability, and adequate cooperation in the outpatient setting. When a broader rehabilitation was required—such as full-mouth treatment, multiple extractions, or prolonged operative time—patients were triaged in advance to management under general anesthesia in an operating room. Compared with a fully cooperative patient, this approach prioritizes completion of essential care through shorter, predictable appointments, with the scope of each visit personalized to the patient’s tolerance and clinical needs.

## 4. Discussion

This study provides a pragmatic and clinically grounded perspective on dental treatment options for non-cooperative patients, integrating real-world sedation data from an Italian setting with evidence from the international literature. Taken together, these findings aim to support a pragmatic stepped-care pathway for non-cooperative patients that is explicitly derived from three data-informed components. As such, structured triage should distinguish patients suitable for outpatient sedation from those requiring planned operating-room general anesthesia (GA), based on tolerance, behavioural profile, and anticipated procedural burden. Second, when outpatient sedation is appropriate, care should be intentionally delivered in a stepwise manner with a limited and pre-planned scope per session (e.g., a single quadrant or a restricted number of teeth) to ensure cooperation and maintain safety. Decision-making should therefore incorporate system-level feasibility, including availability of trained teams, monitoring and recovery infrastructure with timely access to GA. In cases of extensive rehabilitation, multiple extractions, or prolonged operative time, GA already scheduled from the outset is preferable to repeated outpatient attempts. In this framework, behavioural strategies, sedation, and GA are complementary components of a single integrated pathway, reinforced by prevention, structured recall, and caregiver involvement [29,30,31,32,33].

### 4.1. General Anesthesia Versus Sedation: Clinical Roles and Limitations

Across both Italian and international studies, a consistent pattern emerges whereby general anesthesia (GA) is preferentially used for patients with severe intellectual disability, profound non-cooperation, or extensive treatment needs. GA allows comprehensive dental rehabilitation to be completed in a single session, providing full control of patient movement and reflexes and maximizing procedural success. In patients with developmental disorders and long-standing difficulties in oral hygiene, GA-based treatment is frequently associated with a higher number of extractions, reflecting more advanced disease at the time of presentation [34,35,36].

However, several studies have highlighted important limitations of a GA-centred approach. Post-GA recall attendance is often poor, with Italian and international reports indicating that fewer than 20% of patients attend regular follow-up visits after comprehensive rehabilitation under GA. As a result, the long-term benefits of a single, extensive intervention are frequently undermined by the re-accumulation of disease, leading to repeated episodes of pain, infection, and, in some cases, the need for subsequent GA [37,38,39].

Sedation, by contrast, offers a less invasive alternative that can be delivered in ambulatory settings and may be suitable for a subset of non-cooperative patients, particularly those with severe dental anxiety or milder forms of disability. In our clinical series, appropriately selected patients completed planned procedures safely under oral or intravenous benzodiazepine-based protocols, without conversion to GA or major complications. From a system perspective, sedation may reduce pressure on operating rooms, which are costly and often limited in availability. Nonetheless, sedation has inherent limitations. In patients with extreme behavioral resistance or significant medical complexity, sedation may be inadequate, necessitating escalation to GA [19,21].

Moreover, safe sedation requires trained personnel, appropriate monitoring, and organizational structures that, in practice, may not be substantially less demanding than those required for GA.

The spectrum of sedation regimens used across the entire cohort in routine practice is described in Table 4. It must be noted that regimen selection was individualized by the anesthesiology team according to real-time clinical judgment (patient tolerance, comorbidities, airway considerations, and anticipated session duration) and was not protocolized to specific indication subgroups or procedure categories. Therefore, the present retrospective dataset does not support a prescriptive regimen-by-procedure algorithm linking Table 4 to Table 5, and no comparative analysis of regimen choice by indication group or procedure category was performed.

### 4.2. Oral Health Status and Short- Versus Long-Term Outcomes

The literature consistently demonstrates that non-cooperative patients present with poor baseline oral health, characterized by a high prevalence of untreated caries, periodontal disease, and irregular preventive care [40,41]. These factors contribute to frequent presentation with pain, infection, and functional impairment, such as difficulty chewing. Both sedation and GA are highly effective in resolving acute dental disease. Studies report substantial short-term improvements in comfort, mastication, and behavior following treatment, as also reflected in caregiver reports. Both sedation and GA are highly effective in resolving acute dental disease. Studies report substantial short-term improvements in comfort, mastication, and behavior following treatment, as also reflected in caregiver reports [36,42,43,44,45,46,47].

However, long-term outcomes depend critically on sustained preventive care. Persistent challenges—including difficulties with daily toothbrushing, cariogenic diets, polypharmacy, and limited access to routine check-ups—mean that new disease frequently develops after initial treatment, regardless of whether care was delivered under sedation or GA.

### 4.3. Access to Care: The Italian Context in a Global Perspective

In Italy, access to GA-based dental care is largely concentrated in hospital settings located in major urban centers. Many regions lack local specialist services, forcing families to travel long distances and endure waiting times that may extend for several months. Surveys indicate that a substantial proportion of Italian dentists do not routinely treat patients with significant cognitive impairment, citing insufficient training, lack of support staff, and absence of sedation or GA facilities [44,48].

Similar challenges are reported internationally, although countries such as the United Kingdom and several Nordic nations have developed more structured special-care dental services, including dedicated referral pathways and community-based sedation programmes. While essential dental care for certified disabled patients is theoretically covered by the Italian National Health System, practical barriers—including out-of-pocket costs, travel burdens, and administrative complexity—continue to limit access. Preventive oral health is rarely integrated into disability services, and caregivers frequently report a lack of formal training in oral hygiene techniques [36,47].

International experience suggests that the most effective models combine community-based prevention, accessible sedation services, and clearly defined pathways to hospital-based GA for complex cases. In Italy, elements of such models exist but are inconsistently implemented, with limited capacity, variable funding, and a lack of standardized regional pathways [49].

Several limitations of this study should be acknowledged. First, the clinical dataset is relatively small and heterogeneous; therefore, subgroup comparisons should be considered exploratory and were not powered for definitive inference. Second, the retrospective design and reliance on routinely collected clinical documentation limited the availability of standardized outcome measures; consequently, variables such as American Society of Anesthesiologists (ASA) status were inconsistently reported and could not be analysed, and long-term endpoints (e.g., recall attendance, disease recurrence, and durable behavioural adaptation) were not systematically captured. This limitation has important implications for external validity. Without ASA physical status (or an equivalent standardized anesthesiology risk classification), we could not stratify outcomes by baseline medical risk or evaluate whether the observed feasibility and safety profile differed in higher-risk patients. Therefore, our findings should be generalized primarily to settings and patient profiles comparable to those captured in our records—namely, non-cooperative patients selected for outpatient sedation by experienced teams with appropriate monitoring and recovery infrastructure—while caution is warranted when extrapolating to patients with substantial systemic comorbidity (e.g., ASA III–IV) or to services with different anesthesiology pathways and resource constraints. Prospective studies should incorporate structured anesthesiology data capture (including ASA classification and comorbidity indices) to enable robust risk adjustment and clearer applicability across healthcare systems. Third, although procedural completion and short-term safety were consistently recorded, the absence of a parallel control group and the observational nature of the study preclude causal inferences or robust comparative conclusions between sedation and general anesthesia. Fourth, severe dental phobia was defined based on external specialist referral documentation, and standardized psychometric scoring (e.g., MDAS) or a uniform structured DSM-5 interview was not systematically available within our retrospective dataset. Fifth, the single-centre Italian setting may limit generalizability to health systems with different referral pathways, workforce capacity, and access to specialist services; however, situating these findings within the broader international literature enhances their external relevance and highlights common structural barriers faced by non-cooperative patients across settings.

Despite these limitations, the study provides a pragmatic view of sedation-assisted dental care in routine practice, spanning diverse causes of non-cooperation and a broad procedural spectrum, with detailed documentation of sedation protocols and immediate outcomes. In line with published evidence, our findings underscore that non-cooperative patients frequently experience substantial unmet dental needs and restricted access to timely care, often resulting in delayed treatment and avoidable morbidity. From a clinical and policy perspective, these observations support a stepped-care framework in which behavioural strategies, sedation, and general anesthesia are integrated rather than viewed as competing options. Strengthening training in special-care dentistry and sedation, implementing clear regional referral pathways, and embedding preventive oral health within disability services may improve continuity of care. Finally, ensuring equitable access to effective dental treatment for people with disabilities and other non-cooperative patients remains an ethical priority consistent with health-equity principles and the United Nations (UN) Convention on the Rights of Persons with Disabilities.

## 5. Conclusions

Non-cooperative patients—whether due to disability, neurological impairment, or severe dental fear—remain among the most underserved groups in dentistry. This Italian experience suggests that conscious and deep sedation, when delivered by trained teams in appropriate settings, can safely facilitate a meaningful range of dental treatments in selected patients, while general anesthesia remains indispensable for individuals with profound non-cooperation, complex medical needs, or extensive disease. However, our retrospective dataset does not support prescriptive regimen-by-procedure recommendations and was not designed to provide comparative effectiveness evidence versus general anesthesia.

No single approach is universally optimal. High-quality care requires an individualized, stepped strategy that integrates behavioural management, sedation, and general anesthesia rather than treating them as competing alternatives. This framework should be reinforced by strong preventive programmes, structured recall, and active caregiver involvement to reduce crisis-driven care and improve long-term oral health outcomes.

Accordingly, the question of “which approach is best” has no single answer. The most effective pathway combines individualized triage across behavioural techniques, sedation, and general anesthesia; integration of these modalities within an explicit stepped-care model; and sustained investment in prevention, follow-up, and caregiver support. At a systems level, bridging the gap between what is technically feasible and what is practically accessible will require coordinated action in training, infrastructure, referral networks, and equitable funding.

Future studies should adopt prospective, multicentre designs with standardized outcome measures to better define patient selection criteria and to evaluate long-term outcomes, including sustained oral health improvement and service utilization. In parallel, health-services research should assess organizational strategies that expand access to sedation and preventive care and clarify how sedation can be optimally integrated within stepped-care models alongside behavioural management and general anesthesia. If such efforts are pursued, sedation and general anesthesia can move beyond last-minute “rescue” interventions and become predictable components of an organized, inclusive oral healthcare pathway that truly leaves no one behind.

## Figures and Tables

**Table 1:** Demographic characteristics of the analysed sedation sessions.

Variable	Value
Number of analysed sessions	41
Mean age, years (±SD)	32.0 ± 14.0
Age range, years	6–75
Pediatric sessions (<18 years)	14 (34.1%)
Adult sessions (≥18 years)	27 (65.9%)
Male	22 (53.7%)
Female	19 (46.3%)

Data are presented as mean ± SD for continuous variables and n (%) for categorical variables. SD, standard deviation.

**Table 2:** Descriptive characteristics of sedation-assisted dental treatment sessions.

Variable	Value
Total sedation-assisted sessions identified	43
Sessions included in the primary analysis	41
Sessions excluded (IM sedation or general anesthesia)	2
Patients with disability or neurological impairment	20 (48.8%)
Patients with severe dental phobia	21 (51.2%)
Route documented (oral/IV/combined)	39 (95.1%)
Oral sedation	12/39 (30.8%)
Intravenous sedation	18/39 (46.2%)
Combined oral and intravenous sedation	9/39 (23.1%)
Major adverse events	0
Treatment completion rate	41/41 (100%)

Data are n (%) unless otherwise specified. IV, intravenous; IM, intramuscular. Route documented refers to sessions with explicit route documentation (denominator 39).

**Table 3:** Dental procedures performed under sedation.

Procedure Category	Number of Session (n)
Preventive procedures (professional oral hygiene, scaling)	19
Restorative procedures (fillings, endodontic treatments)	12
Surgical procedures (simple/surgical extractions, germectomies)	10
Diagnostic procedures only	0

Procedure categories are mutually exclusive at the session level. Data are n (%) unless otherwise specified. The term “germectomies” refers to the surgical removal of a developing third-molar tooth germ (typically an impacted/unerupted third molar in early development).

**Table 4:** Sedation drugs and adjunctive agents used during analysed sessions.

Sedation Regimen	Number of Sessions (n)
Midazolam alone (oral or IV)	14
Midazolam + propofol	18
Midazolam + propofol + opioid (fentanyl)	2
Midazolam + ketamine	2
Propofol-based sedation without benzodiazepines	1
Other sedation regimens *	4
Total analysed sessions	41

IV: intravenous. * Other sedation regimens included combinations not listed above as separate categories in the primary dataset.

**Table 5:** Subgroup comparison and statistical analysis.

Outcome	Disability/Neurological Impairment (n = 20)	Severe Dental Phobia (n = 21)	Effect/Test
Age, mean ± SD (years)	35.4 ± 12.8	28.6 ± 14.2	Mean diff 6.8 (95% CI −1.7 to 15.3); Welch *p* = 0.115; Hedges’ g ≈ 0.49
Preventive procedures, n (%)	14 (70.0)	5 (23.8)	RR 2.94 (95% CI 1.30–6.66); Fisher *p* = 0.0048
Restorative procedures, n (%)	3 (15.0)	9 (42.9)	RR 0.35 (95% CI 0.11–1.11); Fisher *p* = 0.085
Surgical procedures, n (%)	3 (15.0)	7 (33.3)	RR 0.45 (95% CI 0.13–1.50); Fisher *p* = 0.277
Overall procedure distribution	—	—	Pearson χ^2^(2) = 8.84; *p* = 0.012; Cramer’s V = 0.46

RR compares disability/neurological impairment vs severe dental phobia. Continuous outcomes are reported as mean ± SD. CI, confidence interval; RR, relative risk; χ^2^, chi-square; SD, standard deviation. Procedure categories are mutually exclusive at the session level.

## Data Availability

The data generated and analyzed during this study are not publicly available due to institutional and privacy policies but are available from the corresponding author upon reasonable request.

## Abbreviations

The following abbreviations are used in this manuscript:

GA	General anesthesia
SD	Standard Deviation
RR	Relative Risk
CI	Confidence Interval
ASA	American Society of Anesthesiologists
